# Application-Specific Evaluation of a Weed-Detection Algorithm for Plant-Specific Spraying

**DOI:** 10.3390/s20247262

**Published:** 2020-12-18

**Authors:** Thijs Ruigrok, Eldert van Henten, Johan Booij, Koen van Boheemen, Gert Kootstra

**Affiliations:** 1 Farm Technology, Department of Plant Sciences, Wageningen University and Research, 6700 AA Wageningen, The Netherlands; eldert.vanhenten@wur.nl (E.v.H.); gert.kootstra@wur.nl (G.K.); 2 Field Crops, Wageningen Plant Research, Wageningen University and Research, 8200 AK Lelystad, The Netherlands; johan.booij@wur.nl; 3 Agrosystems Research, Wageningen Plant Research, Wageningen University and Research, 6700 AA Wageningen, The Netherlands; koen.vanboheemen@wur.nl

**Keywords:** deep learning, weed detection, agricultural robotics, weed removal, field test

## Abstract

Robotic plant-specific spraying can reduce herbicide usage in agriculture while minimizing labor costs and maximizing yield. Weed detection is a crucial step in automated weeding. Currently, weed detection algorithms are always evaluated at the image level, using conventional image metrics. However, these metrics do not consider the full pipeline connecting image acquisition to the site-specific operation of the spraying nozzles, which is vital for an accurate evaluation of the system. Therefore, we propose a novel application-specific image-evaluation method, which analyses the weed detections on the plant level and in the light of the spraying decision made by the robot. In this paper, a spraying robot is evaluated on three levels: (1) On image-level, using conventional image metrics, (2) on application-level, using our novel application-specific image-evaluation method, and (3) on field level, in which the weed-detection algorithm is implemented on an autonomous spraying robot and tested in the field. On image level, our detection system achieved a recall of 57% and a precision of 84%, which is a lower performance than detection systems reported in literature. However, integrated on an autonomous volunteer-potato sprayer-system we outperformed the state-of-the-art, effectively controlling 96% of the weeds while terminating only 3% of the crops. Using the application-level evaluation, an accurate indication of the field performance of the weed-detection algorithm prior to the field test was given and the type of errors produced by the spraying system was correctly predicted.

## 1. Introduction

Current agriculture is characterized by its high degree of mechanization, labor efficiency, and the search for ever higher yield [1]. The usage of effective herbicides is one of the key factors to manage weeds in the field [2]. However, the use of herbicides has a significant impact on the environment [2,3]. Site-specific spraying aims to reduce the environmental impact by only spraying parts of the field that contain weeds, instead of the conventional full-field spraying. Site-specific spraying can reduce herbicide usage by up to 70% while maintaining 100% weed control [4,5]. In this paper, we propose and evaluate methods for autonomous robotic weed control with the aim to spray only weeds without harming the crop or spraying the soil.

Crop and weed localization and identification (further referred to as detection) is the very first and crucial step in the process of automated weeding. There is a long history in the development of computer-vision-based weed detection, with research dating back to the eighties. Handcrafted algorithms were used to discriminate weeds from crops [6,7,8,9,10]. Later, handcrafted feature detection was combined with machine-learning algorithms to boost performance [11,12,13,14]. In more recent studies, performance could be further improved by using deep-learning algorithms. These algorithms learn the relevant image features to detect weeds directly from the camera images [15,16,17,18,19,20,21,22,23,24]. 

Regardless of the detection method used, the detection algorithms were always evaluated at the image level [6,7,8,9,10,11,12,13,14,15,16,17,18,19,20,21,22,23,24]. Detection performance was reported in the form of image metrics, such as intersection over union (IoU), precision, recall, and/or mean Average Precision (mAP). However, in robotic weed-control systems, image-based object detection is only the very first step towards a field application. In a field application, the image-based object detection is integrated into a pipeline of image acquisition, processing and actuation as individual nozzle control/site specific operation. Therefore, classification performance at the image level can only serve as a crude indicator of the final performance of the weed-control system. Usually, when driving over the field, the system will process sequences of overlapping images, and the performance of the cumulative detections on the image sequence may differ from the performance on individual images. Furthermore, different types of detection errors will result in different practical effects on the field. For example, a crop plant detected as weed will result in unwanted removal of the crop, whereas a part of the soil detected as a weed, will not directly harm the crop. The current image-level evaluation does not distinguish between these types of mistakes. Ideally, the plant-detection is integrated in a complete system and the whole pipeline, including site-specific application, is evaluated. The evaluation of an integrated robotic weeding system has been done in the past [25,26,27,28,29,30]. However, a field test is time consuming and resource intensive, limiting the number of studies in which the complete system is evaluated and slowing down the development of commercially available weeding robots. To increase the development of plant-specific weeding robots, an image-based evaluation method is available that accurately reflects the in-field performance of the detection system for the application of weed control. 

In this paper, a novel evaluation method is proposed for the application-specific image-evaluation of the plant-detection system. The effectiveness of this method was evaluated by comparing the system evaluation at three levels: (1) On image level, in which common image-based metrics such as precision and recall were used, (2) on application level, in which the effects of the plant-detection system on the robot’s spraying performance were considered, and (3) on field level, in which the plant-detection algorithm will be implemented on an autonomous spraying robot, and the performance of the complete system will be evaluated in the field.

As use case to demonstrate our approach, we focused on the elimination of volunteer potato in a sugar beet field. Volunteer potatoes are leftover potato tubers from previous cultivations, which must be removed to prevent the development of soil-borne diseases like potato cyst nematodes. Furthermore, Dutch law obliges farmers to remove volunteer potatoes to prevent the spreading of *Phytophthora infestans* [31]. Requirements for the robotic system dictated that the elimination rate of the volunteer potatoes should be higher than 95%, while the elimination rate of the sugar beet plants should be lower than 5% [26].

The remainder of this paper is organized as follows. Section 2 describes the materials and methods, including the spraying robot, the weed-detection algorithm, the data acquisition, and the evaluation methods used. In Section 3, the results of the image-level, application-level, and field-level analysis are given. The results are discussed in Section 4 and the work is concluded in Section 5. 

## 2. Materials and Methods

The materials and methods are organized as follows: The spraying robot is described in Section 2.1. In Section 2.2, the data collection is described and finally, in Section 2.3, the experiments conducted and evaluation methods are described.

### 2.1. Autonomous Spraying Robot

**Figure 1:**
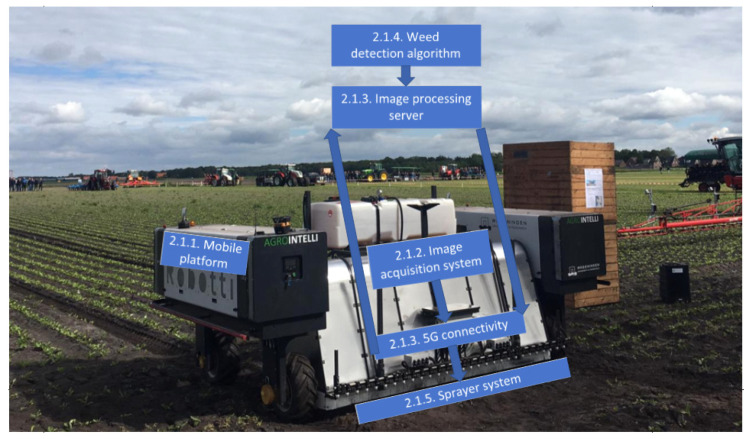
Overview of the robotic precision spraying system. The blocks in the figure highlight the individual components of the robot and show the section number in which the component is described in detail.

Figure 1 shows the robotic system with different components that will be discussed in the next subsections. The robot consisted of a mobile platform (Section 2.1.1) that autonomously navigated through the field, while the image-acquisition system on board the robot took images of the field (Section 2.1.2). The images were sent to a processing server, about 200 km offsite, using a 5G Wireless Wide Area Network (WWAN). On the processing server (Section 2.1.3), the images were processed by the weed-detection algorithm (Section 2.1.4). The locations of the detected plants were sent back to the robot, through the 5G connectivity system. The computer on the robot used the plant detections to control the spraying system, which applied the herbicide glyphosate at the right location (Section 2.1.5).

#### 2.1.1. Mobile Platform

The autonomous Robotti (manufactured in 2019 by Agrointelli) was used as the mobile platform. The four-wheeled robot, built in a catamaran-like setup, consisted of two engine and electronic bays connected by a strong tubular frame and weighed 1850 kg without any implement attached. Mounted to the center of this frame are a standard three-point hitch and PTO-drive. The robot’s 3.15 m wheelbase and design allowed for mounting an implement with the traditional working width of 3.0 m. Power was provided by two Kubota D902 diesel engines and was transferred to the wheels and steering system via a computer-controlled hydraulic system. An integrated task-controller allowed for autonomous operation, following preprogrammed instructions. During the autonomous operation, GPS-based navigation was used based on precise position information from an AgLeader GPS6500 RTK GNSS receiver. The RTK correction signal was provided by 06-GPS, a regional subscription-based correction signal provider. 

#### 2.1.2. Image Acquisition System

**Figure 2:**
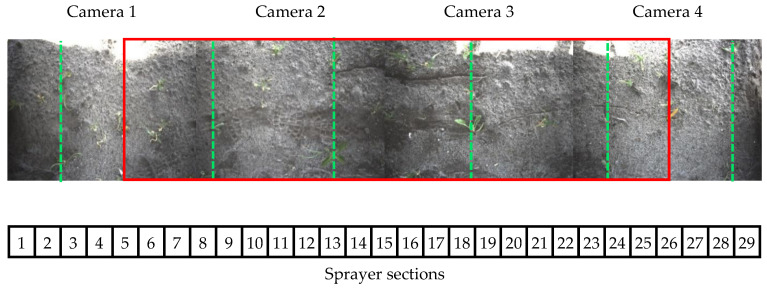
Overview of the camera and sprayer system. The field of view of the four cameras is shown by the four pictures. The field of view of the cameras does not overlap. The locations of the 29 sprayer sections are shown in the numbered blocks below the images. The four cameras together cover six rows of sugar beets, indicated with the green dotted lines. The trial plots only contain four out of the six crop rows. The area of the trial plot is indicated with the red square.

At the rear of the mobile robot, an image acquisition system was attached. The system consisted of a box, with dimensions of 0.91 by 2.80 by 1.05 m (length × width × height), which can be seen in Figure 1. Underneath the cover were four Chameleon 3 CM3-U3-31S4C RGB cameras with a resolution of 2048 by 1536 pixels. Each camera had a field of view of 0.53 m in line with and 0.70 m orthogonal to the driving direction. The field of view of the camera’s next to each other did not overlap. The four cameras together covered six sugar beet rows, shown in Figure 2. While driving, the cameras were triggered approximately every 25 cm, resulting in an overlap of 62% in the driving direction. With this overlap in the driving direction, each plant is seen by the camera up to three times. While blocking all exterior light from the outside, the inside of the box was covered in tinfoil and was equipped with LED-light strips. The LED-light strips generated a white diffuse light of 6500 K. At the bottom, the box was outfitted with black rubber curtains to prevent ambient light from entering.

The camera system was equipped with a custom-built low-profile Asus computer consisting of an Intel HD77DF motherboard, Intel Core i7–3770T CPU, and 8 GB DDR3 RAM. Each camera was connected to the computer via a USB3.0 network hub.

#### 2.1.3. Image Processing Server

To prevent the use of expensive computing hardware with a high power consumption onboard the robot, the image processing by the weed-detection algorithm was outsourced to a remote server. A 5G network was used to ensure a reliable and high-speed wireless connection. A Huawei 5G transceiver was mounted at a 15 m high telecom mast on the Valthermond university farm, within 300 m from the experimental plot. In the field, a Sony prototype smartphone facilitated a connection to the 5G network. The prototype smartphone was connected to the camera vision system via USB3.0. In line with Dutch legislation, only limited bandwidth between 1800 and 2100 MHz could be used. The uplink carrier aggregation principle was applied to optimize the use of the available bandwidth. This setup allowed for a maximum upstream speed of 10 Mbit/s per connection. By splitting the data into smaller packages, and sending the data over multiple connections simultaneously, a maximum upstream speed of 120 Mbit/s was achieved. Using this 5G connectivity system, the images were sent to a KPN data processing server in The Hague, approximately 200 km from the experimental field. The server had 128 GB of RAM and an NVIDIA Tesla M10 GPU, connected to the internet via a 10 GB/s connection. After processing, the pixel locations of the detected plants were sent back to the robot. The time between sending the image data and receiving the plant locations was approximately 250 ms. 

#### 2.1.4. Weed Detection Algorithm

Deep learning object detection algorithms outperform handcrafted algorithms in the agricultural domain [19,21,24]. Therefore, in this research, a deep learning algorithm was used for the weed detection task. Considering the use case of an automated volunteer potato removal robot, an accurate algorithm that runs in real-time was needed. Therefore, the YOLOv3 object detector developed by Redmon [32] was used. This YOLOv3 object detector is almost as accurate as state-of-the-art object detectors such as Faster RCNN [33] and RetinaNet [34], but much faster [32]. In the following sections, the architecture of the YOLOv3 algorithm will be described, the procedure for training the algorithm will be given and the data used for training will be presented. 

##### Algorithm Architecture 

**Figure 3:**
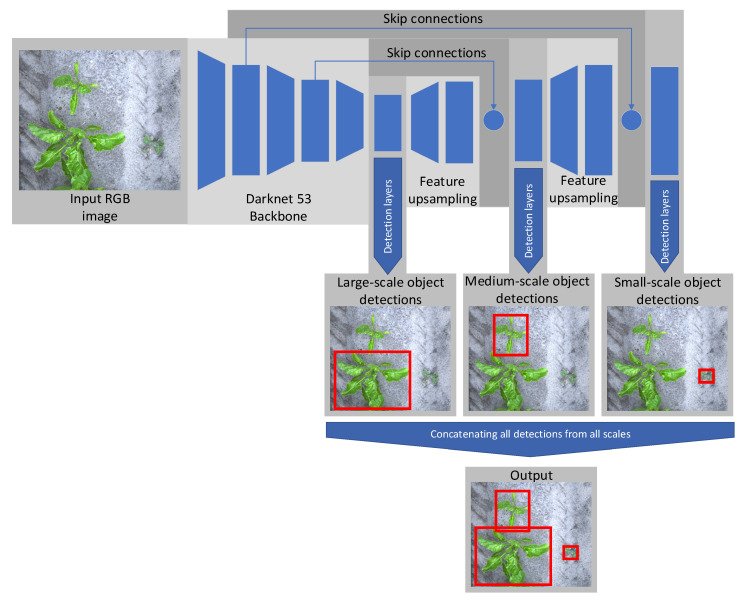
Schematic overview of the YOLOv3 object-detection network applied to crop and weed detection.

In our research, we used the open-source YOLOv3 implementation of Jocher et al. available at [35]. Figure 3 illustrates the YOLOv3 network. The backbone of the object detector is Darknet 53, a fully convolutional feature extractor with 53 convolutional layers. The backbone generates a feature map, which is an abstract description of the input image. The feature map is used by the detection layers to make object predictions. To make the network more robust to variation in scale, predictions at three different scales are made, using feature maps with different resolutions. The large-scale feature map, generated by the Darknet-53 backbone, has a resolution 32 times smaller than the original image. The medium-scale feature map is created by upsampling the large scale feature map by a factor of two, using skip connections to lower layers in the backbone. This upsampling is repeated one more time to create the small-scale feature map. The three feature maps are used by their respective object-detection layers. The object-detection layers always predict three bounding boxes for each cell in its respective feature map. The output vector for each prediction has seven values. The first four values represent the location and size of the bounding box, the fifth value represents the objectness score, a confidence estimate for the bounding box prediction and the sixth and seventh value are the class confidence for the sugar beet and potato class respectively. By multiplying the objectness with the class confidence, the object confidence is computed.

The detections from all three object detection layers are concatenated together. Most of these detections have a low object confidence and overlap with other detections. To filter these detections, first, a threshold is placed on the object confidence. Detections with a confidence lower than 0.001 are removed from the detection pool. After this first filtering step, non-maximum suppression (NMS) is applied to remove overlapping predictions that attempt to predict the same object. NMS starts by sorting the detections based on their confidence. The detection with the highest confidence is selected, and the IoU with other detections from the same class are calculated. Detections with an IoU higher than 0.5 but with a lower object confidence than the overlapping detection are removed from the detection pool. This process is repeated until there are no detections from the same class with an IoU higher than the given IoU threshold. 

##### Training Procedure

During training and inference, the default hyperparameters, reported by [35] were used. To improve the generalization of the plant-detection algorithm, implicit regularization was used. Prior to training, the model was initialized with weights obtained from pre-training on the COCO dataset [36]. To make the algorithm robust to variation in scale, the algorithm was trained on multiple resolutions between 320 × 320 pixels and 608 × 608 pixels. To further improve the robustness to variation in pose and illumination, the data augmentation parameters optimized by [35], listed in Table 1, were used. 

After training, the detection algorithm was put into inference mode, disabling the data augmentation, and implemented on the data server. During inference, images were fed into the detection algorithm with a resolution of 416 × 416 pixels. Detected plants and their locations in image coordinates were sent back to the spraying robot. 

##### Training Data

The training data used consists of two parts. Part one is specifically acquired with the camera system of our weed spraying robot. To further augment our training dataset we added part two, which consists of images from other unpublished volunteer potato detection projects we did in the past. The complete training dataset has 2260 images in total, containing a total of 6383 sugar beet and 1709 potato annotations. 

**Figure 4:**
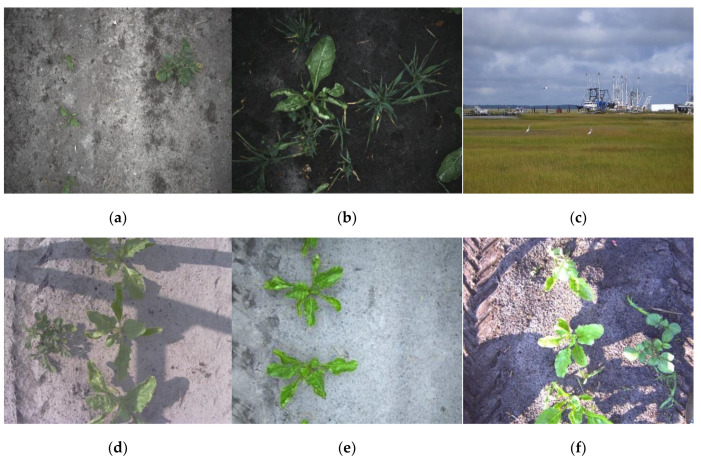

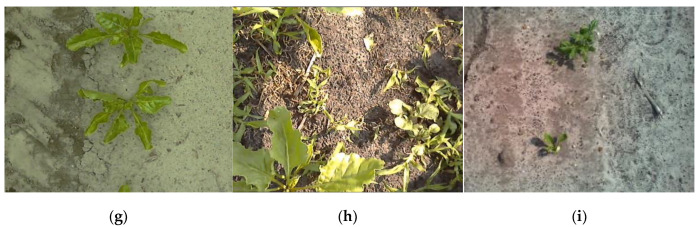
Example images from the training dataset. From all the dataset parts (**a**–**i**) one example image is given. Note the broad variation in lighting, growth stages, soil, presence of weeds and barely, camera quality, and scale.

In spring 2019, sugar beets were sown with a 0.5 m inter-row distance and 0.19 m intra-row distance at the Valthermond university farm. In the same field, potato tubers were manually planted at random locations to simulate volunteer potatoes. On 20 May 2019, images were acquired in this field using the image acquisition system of our weed-spraying robot described in Section 2.1.2. Class imbalance was reduced by only selecting images containing at least one potato plant, resulting in a total of 280 annotated images. Later in the season, at the same farm, using the same protocol, a new field with sugar beets and potatoes was planted to specifically facilitate this research. In this field, on 9 August 2019 more images were collected and only the images containing at least one potato plant were selected and annotated, resulting in a total of 228 annotated images. A technical overview of this dataset is given in Table 2, row a and b. An example of the images from the dataset is given in Figure 4a,b. 

To increase the size of the training dataset, images from other weed detection projects were used. These images are acquired with different acquisition systems and cameras ranging from webcams to industrial cameras, on widely different fields containing crops in different growth stages and under varying illumination conditions. Additional to the sugar beets, some fields also contained barely, which is commonly used in the Netherlands to prevent sandblast damage to the crops. Furthermore, to prevent the object detector from having a high number of false positives on objects not present in the plant datasets, we added a sample of 250 images from the COCO 2017 test set [37] to serve as hard negatives (Table 2 row c and Figure 4c). An overview of the complete training datasets is given in Table 2 and a sample of images is given in Figure 4. 

#### 2.1.5. Sprayer System

The sprayer system consisted of a 2.8 m wide spraying boom, electrically powered pressure pump, and tank for holding the water-herbicide mix. On the sprayer boom, which was under constant pressure, 29 single-outlet nozzle bodies fitted with AgroTop SpotFan 40–03 spraying nozzles were mounted at a distance of 9.5 cm from each other (shown in Figure 2). Operating at 20 cm from the ground, these nozzles sprayed a section of about 10 cm in width. The nozzle bodies were built to be Pulse-width modulation controlled and could be switched on and off individually in 10 ms by a central controller. During the experiments, a glyphosate-herbicide mixed with water was used to spray the potato plants.

Upon receiving the plant locations from the image processing server, the onboard computer converted these plant coordinates in the image frame to locations in the robot coordinate frame. The conversion was based on the assumption of all pixels in the image of all four cameras having a fixed size and a fixed location relative to the robot. The onboard computer sent the coordinates of the potato and the image acquisition time to the spraying controller. The controller of the spraying boom could handle up to 5 detections per second. Each detection contained the position, height, and width of one bounding box. Using the RTK-corrected GNSS location obtained from the Robotti, the spraying-controller calculated the location of the nozzles relative to the detected plants. If a weed (partly) overlapped with a nozzle section, the nozzle was activated. If a detected weed (partly) overlapped with multiple nozzle sections, multiple nozzles were activated. Note that the spraying system needed a minimum of only one detection in one image to activate a spraying section. The spraying system did not account for multiple detections from overlapping images. 

### 2.2. Data Collection

**Figure 5:**
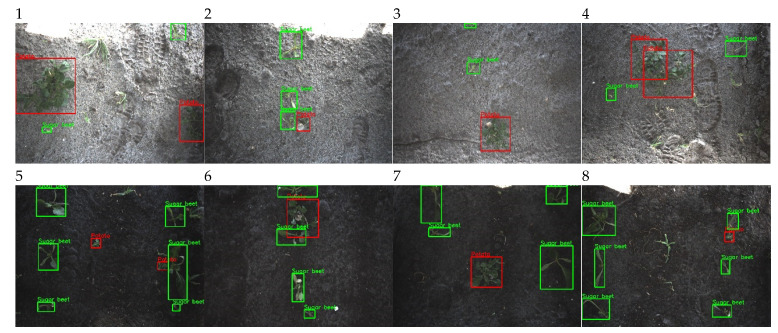
Image sample from the test dataset. One sample from each plot is given. The green boxes are the annotations for the class sugar beet and the red boxes represent annotated volunteer potatoes.

To test the complete robotic system, the field described in Section 2.1.4 was used. In another part, of the field than used for the training dataset, eight trial plots were created for testing the system. Each trial plot was 2 m wide and 12.5 m long, consisting of four sugar beet rows, shown in Figure 2. The trial plots were distributed across different soil types within the same field. Four plots were placed on peaty soil, and the four other plots were placed on more sandy soil. Furthermore, half of the trial plots were additionally seeded with barley (80 kg/ha), as is common practice in this part of the Netherlands to prevent sandblast damage to the crops. The robotic spraying system was used to perform a live field test spraying volunteer potato plants on 23 August 2019. The images acquired by the camera system during the field test were stored resulting in approximately 200 images per plot. All images were manually annotated and used for testing the detection algorithm on both the image level and the application level. This dataset is further referred to as the test dataset. The technical overview of the dataset is given in Table 3 and a sample of images from the dataset is given in Figure 5.

### 2.3. Evaluation Methods

Three aspects of the system were evaluated on the test dataset. In Section 2.3.1 the method to assess the performance of the plant-detection network at the image level is described. The method to assess the performance on the application level is described in Section 2.3.2. Finally, Section 2.3.3 describes the performance evaluation of the autonomous robotic spraying system at the field level. 

#### 2.3.1. Image-Level Evaluation

To evaluate if a prediction of the plant-detection algorithm was correct, the intersection-over union (IoU) between the predicted bounding box and the ground-truth bounding box was used. If the IoU was larger than the threshold of 0.5, the prediction was marked as a true positive (*TP*). If the IoU was lower than the threshold, the detection was marked as a false positive (*FP*). Furthermore, if a ground-truth bounding box did not have a corresponding network prediction with an IoU larger than the threshold, it was marked as a false negative (*FN*). Based on the *TP*, *FP*, and *FN*, the *precision* and *recall* were calculated. The precision is the percentage of network object detections that are correct, see (11) The recall is the percentage of successful object detections, see (22).

(1) $$Precision=\frac{TP}{TP+FP}$$

(2) $$Recall=\frac{TP}{TP+FN}$$

#### 2.3.2. Application-Level Evaluation

The application-level evaluation analyses the crop and weed detections on the plant level and in the light of the spraying decision made by the robot. It is important to note that the spraying robot performs the detection on a sequence of overlapping images. Due to the 62% overlap in the images, a plant can be observed up to three times. The spraying system was not optimized for handling overlapping images and greedily triggers the sprayer if, in those three observations, the plant is at least once identified as a weed. Furthermore, the sprayer sections are already triggered if at least a part of the section contains a potato plant. Therefore, an accurate estimation of the dimensions of the potato plant is not needed. Based on these technical system properties, five rules for the application-level analysis are defined:

1. Each plant counts as one detection, be it a *TP*, *FP*, or *FN*, even though it can be seen in multiple images.
  - If a potato plant is at least once correctly detected, it is counted as a *TP*, because this will result in a correct spraying action.
  - If a sugar beet is at least once wrongly detected as a potato, it is counted as a *FP*, because this will result in an incorrect spraying action, terminating the sugar beet.
  - If a background object, such as soil or a rock, is detected as a potato, it is counted as a *FP*, because this will result in an incorrect spraying action, wasting spraying chemicals.
2. If a potato detection covers multiple potato plants, each of these potato plants is a *TP*, as it will result in correctly spraying the potato plants.
3. If a potato detection overlaps with one or multiple sugar-beet plants, each of these sugar-beet plants is counted as a *FP*, as it will result in spraying and terminating the sugar-beet plants.
4. If a potato plant is detected as multiple smaller potato plants, it is only counted as one *TP*, because the resulting spraying action will only kill one potato plant.
5. If a sugar beet plant is detected as one or multiple potato plants, it is only counted as one *FP*, because the resulting spraying action will only kill one sugar beet plant.

#### 2.3.3. Field-Level Evaluation

**Figure 6:**
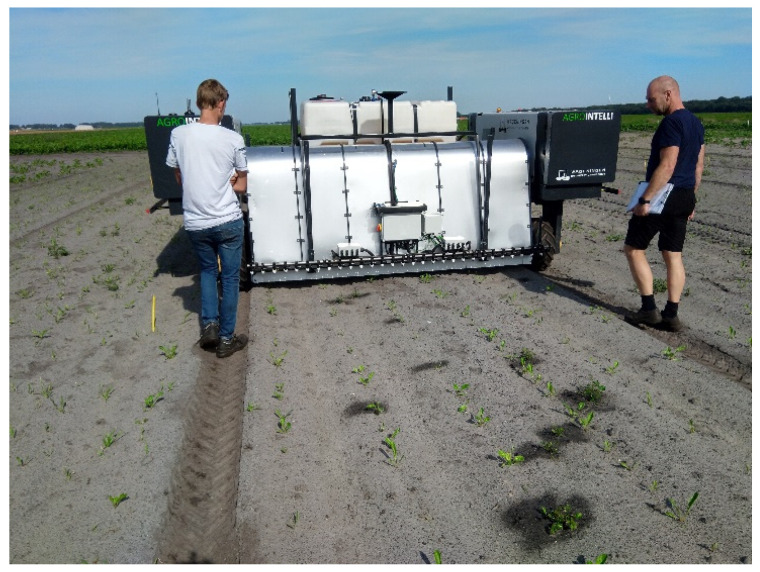
Experts counting the sprayed potato plants and sprayed sugar beet plants.

To quantify the performance of the precision-spraying robot, first, all potato plants and sugar beet plants within the plot were counted by experts of the Valthermond experimental farm. Then, the system was used to spray volunteer potatoes in the test field with a mix of water and a glyphosate-herbicide. As the soil and weather were warm and dry, the expert could easily see the spots and plant parts that were sprayed (Figure 6). The experts counted the sprayed potato plants and sprayed sugar beet plants. Additionally, the experts qualitatively assessed if a sprayed sugar beet was close enough to a sprayed potato plant, to assume that the sugar beet was sprayed due to the coarse resolution of the spraying system. 

## 3. Results

In this section, the results of the evaluation on the three different levels is provided. Section 3.1 provides the image-level evaluation. The application-level evaluation is given in Section 3.2 and the field-level evaluation is presented in Section 3.3.

### 3.1. Image-Level Evaluation

**Figure 7:**
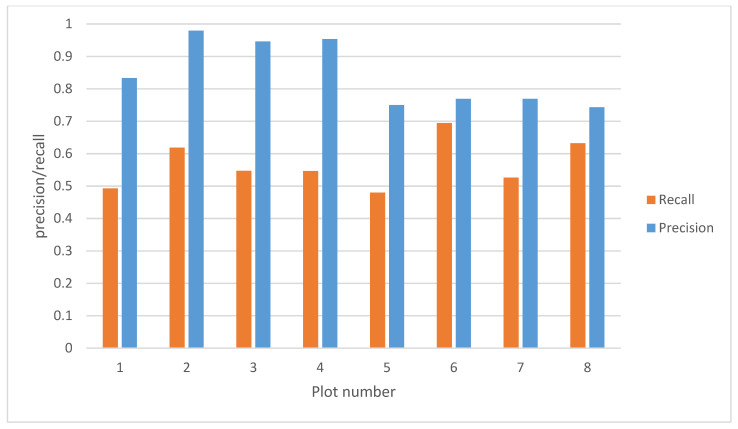
Image-level evaluation results of the plant-detection algorithm on the test dataset.

The precision and recall of the potato class are given in Figure 7. The recall for the potato class is on average 0.57. This means that only 57% of the potato annotations present in the dataset are correctly detected, which is low relative to our requirement stating that 95% of the potatoes need to be sprayed. The precision is on average 0.84. This means that 84% of the model detections for the potato class correspond to a ground truth annotation of a potato. However, it does not tell if this means that a sugar beet is detected as a potato. Using the precision, it cannot be validated if the second requirement, demanding less than 5% of the sugar beets to be terminated, is met. The precision simply does not tell whether a false detection results in the killing of a sugar beet or spraying of bare soil. 

**Figure 8:**
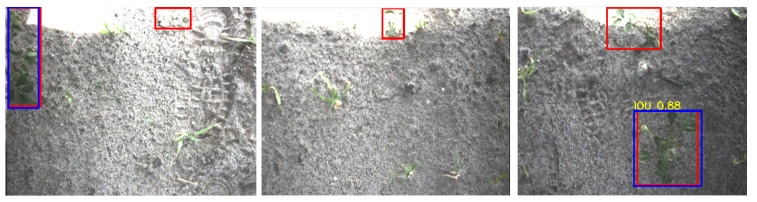
Potato plants are not detected in bright parts of the images. The red rectangles represent the ground-truth annotations and the blue rectangles represent the detections.

Upon visual inspection of the potato detections, it can be seen that the detection algorithm poorly detects potatoes near the upper edge of the image, explaining the low recall. In this region of the image, light sometimes passes underneath the cover of the camera system, causing over-illumination. Examples of this scenario are given in Figure 8. Although the potatoes are not detected in these images, the camera system has a 62% overlap in the driving direction and thus has two more observations to correctly detect the potato. Therefore, the image level evaluation gives an underestimation of the recall of the potato class. 

**Figure 9:**
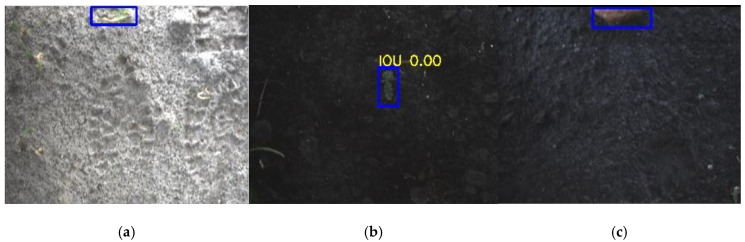
Background objects detected as potatoes. The blue rectangles represent the detections. In panel (**a**,**b**) an unknown weed is detected as a potato and in panel (**c**) a rock is detected as a potato.

Furthermore, background objects, such as unknown weeds and rocks are sometimes identified as a potato, lowering the precision. This is especially the case in plot 5–8. Plot 5–8 were located on peaty soil and contained more small weeds than the other plots Examples of this scenario are given in Figure 9. Although these detections are wrong, the crop will not be damaged by spraying those objects, therefore these errors have only a limited effect on the practical applicability of the system. The image-level analysis does not give insight in this type of error. 

### 3.2. Application-Level Evaluation

**Figure 10:**
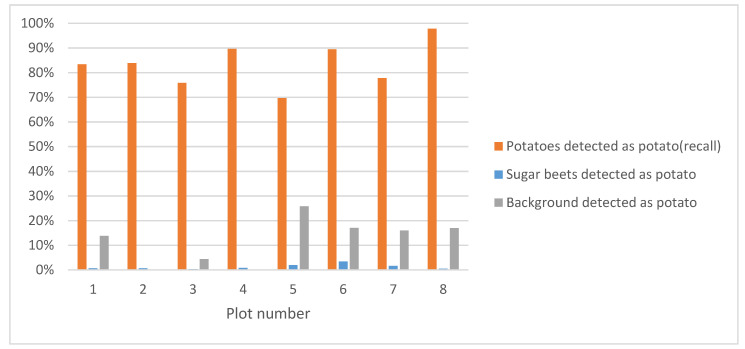
Application-level evaluation results of the plant-detection algorithm on the test dataset.

Contrary to the image-level evaluation, the application-level evaluation does take the practical implications of the image detections into account. The application-level evaluation performance is given in Figure 10. Using the application-level evaluation, 83% of the potato plants were correctly detected. This means the requirement to identify and spray at least 95% of the potato plants is not met. Only 1% of the sugar beets were wrongly classified as potatoes. This is better than our requirement, which states that a maximum of 5% of the sugar beets can be killed. Furthermore, 12% of the potato detections were actually background. Though these detections are incorrect, their consequences are limited as this only resulted in the unneeded spraying of the background, but this did not damage the crop. 

Figure 10 shows a large difference between the plots. In plots 5–8 more false positives were detected than in plot 1–4. Plot 1–4 had sandy soil, whereas plot 5–8 had a more peaty soil. The peaty soil had more small weeds present, which were wrongly detected as potato plants. Furthermore, plots 1, 4, 5, and 8 contained barely. Because barely was also present in the training dataset, the detection algorithm could handle the presence of these objects and correctly detected the barely as background.

**Figure 11:**
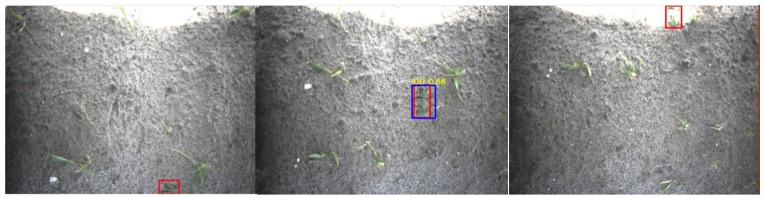
A potato plant was seen three times and detected only once. The red rectangle represents the ground-truth annotations and the blue rectangle represents the detection. Despite the volunteer potato being present in multiple images, this single detection would still result in a correct spraying action. The potato detection was therefore counted as a true positive (*TP*).

**Figure 12:**
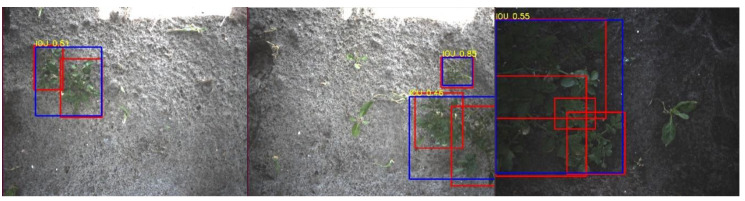
Three examples of multiple potato plants detected as a single potato plant. The red rectangles represent the ground-truth annotations and the blue rectangles represent the detections. The detection resulted in a correct spraying action by the robot, hitting the potato plants. These plants are counted as a *TP*.

**Figure 13:**
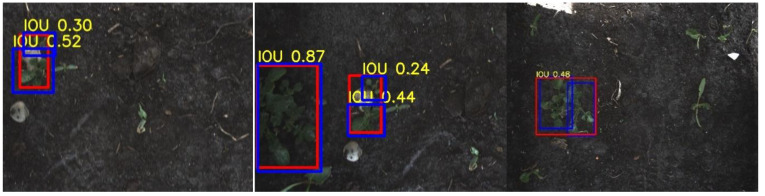
One example of a potato plant detected as two potato plants. The red rectangle represents the ground-truth annotation and the blue rectangles represent the detections. The multiple detections resulted in a correct spraying action. This single potato plant (red rectangle) was therefore counted as one *TP*.

When comparing the image-level evaluation to the application-level evaluation, it can be seen that the recall of the potato class on the application-level is 26 percent point higher. This can be attributed to the following three cases:

Case 1. A single potato plant was seen multiple times, due to overlap of the images of approximately 62% in the driving direction. In some images, the plant detection might be false, especially when a plant was only partially visible. However, if the potato plant was correctly detected at least once in the images from the multiple viewpoints, it would result in a correct spraying action. This plant detection was therefore considered a *TP* in the application-level evaluation, increasing the *TP* rate while reducing the *FP* rate. Examples of this case are given in Figure 11.
Case 2. Multiple smaller potato plants were detected as a single big potato plant. Evaluated at the image level, this is incorrect. However, in the application-level evaluation, this is counted as a true positive, increasing the recall. Examples of this case are given in Figure 12.
Case 3. A big potato plant was identified as multiple smaller plants. This still resulted in a correct spraying action. The application-level evaluation counts this case as a *TP*. An example is given in Figure 13.

### 3.3. Field-Level Evaluation 

**Figure 14:**
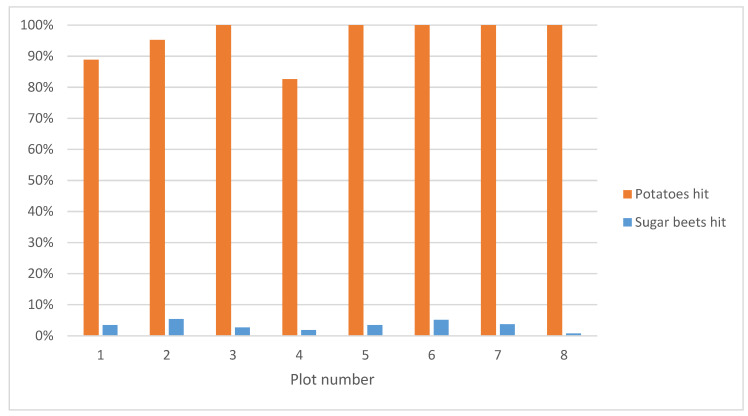
Percentage of potatoes and sugar beets hit by the autonomous spraying system in the test field.

Experts counted the sprayed potato and sugar beet plants. Results of the counting are shown in Figure 14. In the figure, it can be seen that on average, 96% of the potatoes were correctly sprayed and that only 3% of the sugar beets were hit. With these results, the system meets the requirements, which dictated that the elimination rate of the volunteer potatoes must be higher than 95%, while the elimination rate of the sugar beet plants must remain below 5% [26]. 

The experts also counted sugar beets close to a sprayed potato plant, shown in Table 4. It was assumed that these plants were sprayed because of the coarse resolution of the spraying system. On average 50% of the terminated sugar beets were hit because of the coarse resolution of the sprayer. This indicates that the system can be improved by increasing the sprayer resolution. 

When comparing the field-level evaluation to the application-level evaluation, it can be seen that the application-level evaluation has a 13 percent point underestimation for the recall of the potatoes. This is probably caused by the course spraying resolution of the sprayer boom. The sprayer sections are triggered if at least a part of the section contains a potato plant. This course spraying action will also kill undetected potato plants close to other, detected potato plants. Additionally, 2 percent point more sugar beets are hit by the sprayer than predicted with our application-level evaluation. This is partly caused by the course spraying action of the spraying boom. When a potato is detected that overlaps with a sugar beet, it will still trigger a spraying action, killing the sugar beet in the process. From all the sugar beets hit, 50% was classified as close to a potato plant by the evaluation experts. This indicates that 50% of the damaged plants is caused by the limited resolution of the sprayer. 

## 4. Discussion

The discussion is structured as follows: In Section 4.1 the three different evaluation methods are compared with each other. Section 4.2 compares our result with relevant literature and in Section 4.3, the limitation of our methods is discussed and improvements for further work methods are proposed. 

### 4.1. Comparing the Evaluations at Three Different Levels

There is a large difference between the image-level evaluation and the field-level evaluation. The image-level evaluation reported a recall of 57% while a recall of 96% was achieved in the field test. Furthermore, the image-level evaluation did not provide insight in the number of crop plants damaged. 

The robot uses up to three images of the same plant to determine a spraying action. A weed needs to be detected only in one image to get sprayed. This method solves many of the false negatives that we observe on the image-level. These false negatives mainly appear near the borders of the images, where the plants are only partially visible and where there is over-exposure. The down-side of this method, however, is that there is a risk of getting more false positives, as a single false weed detection will result in a spraying action. 

The application-level performance better matches the field-level performance than the image-level performance, and can therefore be considered as a better indication for the performance of a weeding robot. However, the application-level evaluation can be further improved. The effect of the course spraying resolution is not taken into account, therefore the application-level evaluation underestimates the number of crop and weed plants sprayed. To predict the field performance more accurately, we recommend to include the effects of the spraying resolution in the application-level evaluation method. 

### 4.2. Comparison with Related Work

In the test field, at the image level, we achieved a precision of 84.3% and a recall of 56.7%. This performance at the image level is lower than the performance reported in [13,17,18,38]. The work of [13], achieved a recall of 94% at a precision of 98% and for the weed class. The paper of [17] reports a precision of 99.6% and a recall of 96.3% and the study of [18] reports a precision of 95% and a recall of 89% on the class weed. The work in [38] reported a precision of 72.7% and a recall of 95.3% for the detected weeds in a sugar beet field. These results cannot be compared directly to our results, as a different dataset, with different illumination, crop-growth stages, and soil type was used. In these papers, a pixel-wise semantic-segmentation dataset was used, whereas our method was designed for object detection. For the same reason, we could not directly apply and compare our method to this dataset. 

According to our application-level performance, 83% of the potato plants are correctly detected, which is much higher than reported by the image-level analysis, but still too low relative to the requirement of 95% of the potato plants being identified and sprayed. Only 1% of the sugar beets were wrongly classified as potatoes. Furthermore, 12% of the potato detections actually belonged to the background. Only damaging 1% of sugar beets is within our requirement that only 5% of the sugar beets can be damaged. The performance on the application level cannot be compared to other literature as there is to our knowledge no literature available that performs an application-level evaluation.

Our integrated spraying robot successfully controlled 96% of the volunteer potatoes and killed only 3% of the sugar beets. This is within the requirements set for an economically feasible solution [26]. Additionally, the field test performed better than the state-of-the-art volunteer potato spraying machine of [26] which controlled only 77% of the volunteer potatoes, terminating 1% of the sugar beet plants in the process. 

### 4.3. Limitations and Future Improvements

Currently, the application-level performance is assessed using a visual inspection. Inter and intra operator variation in this inspection was not taken into account. For consistency and faster evaluation, it would be better to implement the application-level evaluation in an automated script. Or to evaluate inter and intra operator variation. The same holds for the field evaluation. During the field evaluation, the effect of the spraying actions is manually assessed by field experts. Here the inter and intra operator variation affects the results. For a more consistent analysis of the system, it is recommended to log the spraying actions and automate their evaluation.

The application-level evaluation can be further improved to match the field performance even better by considering the effect of the course spraying resolution. Currently, the application-level evaluation underestimates the number of plants sprayed due to the assumption that the detected bounding boxes in the images can be sprayed perfectly, while in reality, it sprays sections of 10 cm. Also, since a large portion of the wrongly sprayed sugar beets plants in the field test is caused by the course spraying action of the weeding robot, it is interesting to increase the resolution of the spraying system or to use a more sophisticated nozzle control algorithm that explicitly avoids spraying close to a sugar beet and by doing so reduce the number of sprayed sugar beets. 

## 5. Conclusions

In this paper, a novel evaluation method is proposed for the application-specific image-evaluation of a plant-detection system. To test this novel evaluation method, a plant detection algorithm is trained, and its performance in practice is assessed on three levels, image level, application level, and field level. On the image level, our detection system was outperformed by systems reported in the literature. However, integrated on an autonomous volunteer-potato sprayer-system we performed better than the reported state-of-the-art. 

The image-level evaluation cannot accurately predict the field performance of the autonomous spraying system. It does not consider that plants are visible in consecutive images and that the robot needs to detect a potato plant only once to trigger a spraying action. Furthermore, in the image-level evaluation, two or more small potato plants detected as one big potato, or a big plant detected as multiple smaller potato plants are marked as an incorrect detection. However, in practice, this results in a correct spraying action. Furthermore, the image-level evaluation does not provide insight in the type of mistake made by the detection algorithm and does not tell whether a false detection results in the killing of a sugar beet or spraying background. Whereas our application-level evaluation provides insight in these types of mistakes. 

The application-level evaluation that we proposed better matches the field-level evaluation. By summing the detection of the overlapping images and by considering the effects of the detections on the spraying actions, the application-level evaluation gives a more realistic indication of the field performance of a crop/weed-detection system for site-specific spraying. Furthermore, the application-level evaluation is faster and cheaper to perform than the field level experiment, since it doesn’t require the presence of a living crop field nor the availability of a complete spraying robot. Additionally, the application-level evaluation method provides the ability to compare two different detection methods on the same data. And finally, the application-level method provides insight in the type of errors made by the detection algorithm. 

## Figures and Tables

**Table 1:** YOLOv3 data augmentation parameters optimized by [35].

Augmentation type	Description
Translation	±6.8% (vertical and horizontal)
Rotation	±1.1 degrees
Shear	±0.6 degrees (vertical and horizontal)
Scale	±11%
Reflection	50% probability (horizontal-only)
Saturation	±57%
Brightness	±32%

**Table 2:** Technical overview of the training data.

Part	Acquisition Date (s)	Contains Barley	Lighting	Resolution	Number of Images	Number of Sugar Beet Annotations	Number of Potato Annotations
a	20 May 2019	No	Controlled	2048 × 1536	280	1177	440
b	9 August 2019	Yes	Controlled	2048 × 1536	228	738	336
c	2017	No	Natural	640 × 480	250	0	0
d	Summer 2018	No	Natural	1280 × 1024	250	631	193
e	28 May 2018 and 1 June 2018	No	Controlled	2076 × 2076	250	794	161
f	28 May 2018 and 1 June 2018	Yes	Natural	2076 × 2076	250	734	195
g	28 May 2018	No	Controlled	1280 × 720	250	554	93
h	28 May 2018 and 1 June 2018	Yes	Natural	1280 × 720	250	484	121
i	4 May 2019	No	Natural	1280 × 720	250	1271	170

**Table 3:** Technical overview of the test dataset. All images were acquired on 23 August 2019 under controlled illumination. The images are acquired with a resolution of 2048 × 1538 pixels.

Plot Number	Soil Type	Contains Barley	Number of Images	Number of Sugar Beet Annotations	Number of Potato Annotations
1	Sandy	Yes	188	645	71
2	Sandy	No	188	678	76
3	Sandy	No	180	579	64
4	Sandy	Yes	176	365	75
5	Peaty	Yes	180	744	75
6	Peaty	No	184	718	72
7	Peaty	No	176	563	57
8	Peaty	Yes	184	543	87

**Table 4:** Sugar beets hit that were close to a potato plant.

Plot Number	1	2	3	4	5	6	7	8	Average
Percentage sugar beets hit close to a potato	14%	36%	67%	100%	43%	75%	67%	0%	50%
